# Outer Membrane Vesicles of *Helicobacter pylori* 7.13 as Adjuvants Promote Protective Efficacy Against *Helicobacter pylori* Infection

**DOI:** 10.3389/fmicb.2020.01340

**Published:** 2020-06-23

**Authors:** Zifan Song, Biaoxian Li, Yingxuan Zhang, Ruizhen Li, Huan Ruan, Jing Wu, Qiong Liu

**Affiliations:** ^1^Department of Medical Microbiology, School of Medicine, Nanchang University, Nanchang, China; ^2^The First Clinical Medical College, Nanchang University, Nanchang, China; ^3^Key Laboratory of Tumor Pathogenesis and Molecular Pathology, School of Medicine, Nanchang University, Nanchang, China

**Keywords:** adjuvant, *Helicobacter pylori*, outer membrane vesicles, cholera toxin (CT), whole cell vaccine

## Abstract

*Helicobacter pylori*(*H. pylori*), a gram-negative bacterium in the human stomach with global prevalence, is relevant to chronic gastrointestinal diseases. Due to its increasing drug resistance and the low protective efficacy of some anti-*H. pylori* vaccines, it is necessary to find a suitable adjuvant to improve antigen efficiency. In our previous study, we determined that outer membrane vesicles (OMVs), a multicomponent secretion generated by gram-negative bacteria, of *H. pylori* were safe and could induce long-term and robust immune responses against *H. pylori* in mice. In this study, we employed two common vaccines, outer membrane proteins (OMPs) and whole cell vaccine (WCV) to assess the adjuvanticity of OMVs in mice. A standard adjuvant, cholera toxin (CT), was used as a control. Purified *H. pylori* OMVs used as adjuvants generated lasting anti-*H. pylori* resistance for 12 weeks. Additionally, both systematic and gastric mucosal immunity, as well as humoral immunity, of mice immunized with vaccine and OMVs combinations were significantly enhanced. Moreover, OMVs efficiently promoted Th1 immune response, but the response was skewed toward Th2 and Th17 immunity when compared with that induced by the CT adjuvant. Most importantly, OMVs as adjuvants enhanced the eradication of *H. pylori*. Thus, OMVs have potential applications as adjuvants in the development of a new generation of vaccines to treat *H. pylori* infection.

## Introduction

*Helicobacter pylori* (*H. pylori*), a gram-negative bacterium colonizing the mucus layer over the gastric epithelium, has been implicated in the pathogenesis of chronic gastritis and peptic ulcer (Marshall and Warren, 1984). According to a meta-analysis from 2015, approximately 4.4 billion individuals were infected with *H. pylori* worldwide, mostly in developing countries (Hooi et al., 2017). Antibiotic combination therapy, proton pump inhibitor (PPI)-based triple therapy or bismuth-containing quadruple therapy (BQT) are currently used as prevalent and efficient treatments to eradicate the colonization of *H. pylori* (Hu et al., 2020). However, in recent decades, resistance to common antibiotics used against *H. pylori*, such as clarithromycin and levofloxacin, has significantly increased globally (Smith et al., 2019). To determine other effective treatments against *H. pylori* infection, many studies have focused on selecting improved vaccine candidates, including whole cell vaccine (WCV), protein-based vaccine, and synthetic carbohydrate vaccines, etc. (Stubljar et al., 2018). Previously, our research group focused on the outer membrane vesicles (OMVs), a natural secretion of gram-negative bacteria, to assess its potential as a potent immunogen for clinical use. Previous results indicated good performance of OMVs in animal studies, when either utilized as a vaccine or adjuvant (Tan et al., 2018; Liu et al., 2019).

Despite efforts in developing an anti-*H. pylori* vaccine, there are currently no available vaccines in the clinic that provide sufficient protection in humans (Stubljar et al., 2018). *H. pylori* can evade the host immune response via altering cytokine signaling in epithelial and myeloid cells as well as releasing vacuolating cytotoxin A (VacA). Some vaccines are unable to generate sufficient immune responses to qualify for clinical trials (Altobelli et al., 2019; Lehours and Ferrero, 2019). Therefore, more powerful antigens and immunomodulators are needed to invoke a satisfactory immune response (Sutton and Chionh, 2013).

Adjuvants, namely immunomodulators, are small molecules that enhance the immunogenicity of vaccines to improve pathogen suppression and reduce the vaccine dose. While classical adjuvants, such as potassium alum, have been frequently used, subsequent studies have generated novel candidates (Arzeno Carranza, 1950; Sjokvist Ottsjo et al., 2013; Liu et al., 2017; Longet et al., 2019). For anti-*H. pylori* vaccines, cholera toxin (CT) and *Escherichia coli* heat-labile enterotoxin (LT) are widely used as adjuvants to boost the efficacy of mucosal vaccines in mice (Lehours and Ferrero, 2019). However, they both exhibit enterotoxicity, which limit their clinical applications. Similarly, many powerful immunomodulators are not approved for human application due to their toxicity (Talebi Bezmin Abadi, 2016). Probiotics administration might be a useful treatment, but probiotics benefits may be strain dependent (Bruno et al., 2018). Therefore, suitable non-toxic adjuvants, such as oral adjuvant α-galactosylceramide (α-GalCer) and multiple mutant CT (mmCT), are needed to enhance the function of anti-*H. pylori* vaccines (Holmgren et al., 2018; Longet et al., 2019).

OMVs, mainly consisting of outer membrane proteins (OMPs), periplasmic proteins, and lipids, are naturally secreted by all gram-negative bacteria and have diameters of 10–300 nm (Kulp and Kuehn, 2010). Studies have shown that they play various roles in infection development, including biofilm formation, gene transformation, and immune regulation (Jan, 2017). Moreover, multiple OMV-based vaccines have been developed and, been investigated in clinical trials (Leitner et al., 2015; Bottero et al., 2016; Baker et al., 2017; Petousis-Harris, 2018). Additionally, the effectiveness of OMVs as adjuvants was investigated (Tan et al., 2018). In our previous study, we found that OMVs derived from *H. pylori* 7.13 showed protective activity in mice without significant toxicity (Liu et al., 2019). These studies suggest *H. pylori* OMVs can be used as adjuvant candidates. In this study, we used two vaccines based on OMPs and whole cells from *H. pylori* 7.13 to assess the adjuvant potential of OMVs in anti-*H. pylori* vaccines. The results demonstrated that OMVs were more effective adjuvants than CT, regardless of the vaccine type.

## Materials and Methods

### Bacterial Culture and Preparation of OMVs

*H. pylori* strains used in this study were cultured in a *Campylobacter* agar base (Difco Labs, Detroit, Michigan, United States) supplemented with 10% sheep blood (Thermo Fisher Scientific, North Shore City, New Zealand) in a microaerobic environment (5% O_2_, 10% CO_2_, and 85% N_2_) at 37°C. *H. pylori* strain 7.13, a gerbil-adapted strain, was derived from clinical strain B128 and was a gift from Professor Yong Xie at the First Affiliated Hospital of Nanchang University in Nanchang, China. Suspensions of *H. pylori* 7.13 used for immune challenge experiment were prepared from fresh exponential-phase cultures to maintain a high level of viable cells.

OMVs of *H. pylori* strain 7.13 were isolated through ultracentrifugation as previously described (Liu et al., 2016, 2018). In brief, centrifuge 500 mL exponential-phase *H. pylori* strain 7.13 for 1 h at 4,500 × g at 4°C to pellet the bacteria. Then the supernatant was filtered twice through a 0.45-μM Steritop bottle-top filter unit (Millipore, Billerica, MA, United States). The filtrate contained OMVs were centrifuged for 2 hat 20,000 × *g* at 4°C and the pellet was washed with DPBS buffer (Mediatech, Manassas, VA, United States). Then the pelleted OMV samples were resuspended in OptiPrep Density Gradient Medium (Sigma) in DPBS buffer and centrifuged for 24 h at 100,000 × *g* at 4°C in a density gradient (40, 35, 30, 20%). The vesicle fractions were pooled, gently washed three times with DPBS and then dissolved in 1 ml DPBS and stored at −20°C. The yields of OMVs were determined by measuring the protein concentrations using a bicinchoninic acid (BCA) assay kit (Thermo Fisher, Rockford, Illinois, United States), according to manufacturer’s instructions.

To obtain the *H. pylori* inactivated WCV as a negative control, *H. pylori* was gathered in sterile PBS from a confluent growth plate, and the bacterial suspension was diluted to OD 1.5. Formaldehyde solution was then added to 50 mL bacterial suspensions in 250 mL flasks to a final concentration of 0.01 M. The flasks were closed tightly and incubated at 37°C for 2 h with agitation, followed by overnight oscillation at room temperature (25°C). The bacterial suspension was then washed three times with sterile PBS, and the final bacterial pellet was resuspended in sterile PBS and used for the subsequent immunizations. OMPs for immunizations were isolated from *H. pylori* 7.13 as previously described (Carlone et al., 1986). The protein concentrations of WCV and OMPs were measured using a BCA assay kit (Thermo Fisher). Protease-treated OMVs after ultrasonication were used as negative controls in the ELISA analysis. Proteinase K was added to the OMVs after ultrasonication at a concentration of 20 μg/mL and incubated at 60°C for 4 h. The quantification of protease-treated OMVs content was measured via KDO (3-deoxy-D-manno-octulosonic acid) analysis, and commercial *E. coli* lipopolysaccharide (LPS) purchased from Sigma-Aldrich (Saint Louis, MO, United States) was used as the standard (Yokota et al., 2007).

### Ethics Statement

All animal experiments were conducted in compliance with the guidelines of the Animal Welfare Act and related regulations of Nanchang University (Nanchang, China, Approval No. NCDXYD-2018019). All animal work protocols were approved by the animal welfare committee of Nanchang University. The principles stated in the Guide for the Care and Use of Laboratory Animals were followed. All efforts were made to minimize animal suffering during our experiments.

### Animal Experiments

**FIGURE 1:**
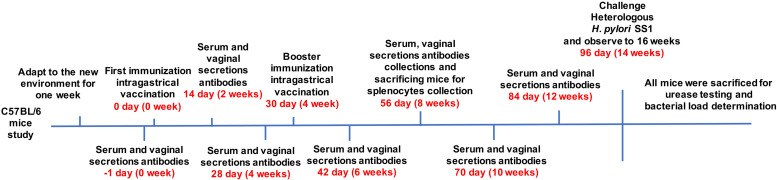
Mice (6 weeks old, 16–22 g) were divided into groups of nine. Mice were immunized with various vaccines, OMPs or WCV by gavage. Booster immunizations were implemented at week 4. Blood samples and vaginal secretions were collected repeatedly and then mice were orally challenged with a lethal dose of *H. pylori* SS1 after booster immunization. The concentration of antibodies was determined at biweekly intervals after first immunization. Animal experiments were performed twice, and the data were combined for analysis.

Female C57BL/6 mice (6 weeks old, 16–22 g) were purchased from the Laboratory Animal Science Center of Nanchang University. After acclimatization to the new environment for one week, the mice were divided into 10 groups. Each cohort contained nine mice and received the first vaccine administration by gavage (0 day). The selected immunogens and dosages administered to each group are described in Table 1. *H. pylori* strain 7.13 WCV consisted of 10^9^ inactivated cells per mouse. CT (Sigma-Aldrich, St. Louis, Missouri, United States) was suspended in 200 μL PBS buffer. PBS alone (10 μL) group served as the negative control. Blood samples and vaginal secretion antibodies were collected one day prior to and 2, 4, 6, 8, 10, and 12 weeks following the first immunization through orbital sinus puncture and flushing five times with 0.1 mL PBS. Subsequently, the soluble fractions of serum and vaginal secretions were obtained by centrifugation. Booster immunizations were performed at week 4 with corresponding antigens by intragastric vaccination. Splenocytes were collected by sacrificing mice at week eight for cytokine detection.14 weeks after the first immunization, all remaining mice were challenged with 10^9^ colony-forming units (CFU) of heterogeneous *H. pylori* SS1 in 20 μL PBS containing 0.01% gelatin (BSG buffer) via the oral route and monitored until week 16. Finally, all mice were sacrificed, and their gastric tissues were harvested for urease tests and bacterial load determination. The immunization and challenge schedules are described in Figure 1. All animal experiments were performed twice, and the data gathered for analysis.

**TABLE 1:** Vaccine formulation strategy for immunization using H. pylori OMVs*.

Group of 9 mice	Immunogen and Dose (μg/one mouse)
1	OMP(200)+OMV(10)
2	OMP(200)+OMV(5)
3	OMP (200)+CT (10)
4	OMP (200)
5	WCV+OMV(10)
6	WCV+OMV(5)
7	WCV+CT(10)
8	WCV
9	OMV(100)
10	PBS control

*6-week-old female C57BL/6 mice as animal model was selected in this study and immunized with oral routes.

### Enzyme-Linked Immunosorbent Assay (ELISA)

To measure the antibody titers in mice blood samples and vaginal secretions ELISA was used as previously described (Liu et al., 2018). For antigen coating, OMPs, intact OMVs and protease-treated OMVs from *H. pylori* 7.13 were suspended in 100 μL sodium carbonate buffer (pH 9.6) and 1 μg of suspension per well was added to 96-well flat plates (Nalge Nunc Inc., Naperville, Illinois, United States) and incubated overnight at 4°C. Purified mouse immunoglobulin (Ig) isotype standards (IgG, IgG_1_, IgG_2__c_, IgA; BD Biosciences, Billerica, Massachusetts, United States) were prepared in triplicate and diluted 2-fold (0.5 μg/μL). After washing the plates three times with PBST (PBS containing 0.1% Tween 20), the plates were blocked with 2% BSA solution at room temperature for 2 h. Subsequently, 100 μL of each sample was added at different dilutions to their respective wells in triplicate, and the plates were incubated for 1 h at room temperature. Biotinylated Goat anti-mouse antibodies IgG, IgG_1_, IgG_2__c_, and IgA (Southern Biotechnology Inc., Birmingham, AL, United States) were added to each well after washing the plates with PBST three times. Streptavidin alkaline phosphatase conjugate (Southern Biotechnology Inc.) was added, and substrate p-nitrophenylphosphatase (Sigma-Aldrich) in diethanolamine buffer (pH 9.8) used to develop the wells. The absorbance 405 nm value was measured on an automated ELISA plate reader (model EL311SX; Biotek, Winooski, Vermont, United States). Standard curves of each antibody isotype were constructed for each Ig subclass concentration, and a log–log regression curve was calculated from at least four-fold dilutions of the isotype standards.

### Cytokine Detection in Mouse Splenocytes

Mouse splenocytes were harvested 4 weeks after booster immunizations, followed by stimulation with 6 μg/mL OMPs isolated from *H. pylori* 7.13 for 24 h, according to our previous report (Liu et al., 2008). Subsequently, supernatants of splenocytes were collected, and splenic cytokines of the stimulated cells were measured by corresponding ELISA kits. Briefly, monoclonal anti–interferon-γ (anti- IFN-γ), anti-IL-4, anti-IL-13, anti-IL-17, and anti-IL-12 (p40) antibodies (BD Biosciences, Mountain View, California, United States) were coated onto 96-well plates. Next, the samples were blocked with PBS containing 1% BSA, added to triplicate wells, and incubated overnight at 4°C. Then, the wells were washed and incubated with biotinylated monoclonal anti–IFN-γ, anti-IL-4, anti-IL-13, anti-IL-17, and anti–IL-12 (p40) antibodies (BD Biosciences, Billerica, Massachusetts, United States). Horseradish peroxidase (HRP)–labeled anti-biotin antibody (Vector Laboratories, Burlingame, California, United States) was then added. To develop the reaction, 3,3′,5,5′-tetramethyl-benzidine (Moss Inc., Pasadena, California, United States) was added, which was stopped with 0.5 N HCl. Finally, a standard curve was generated based on mouse recombinant (r) IFN-γ, IL-4, IL-13, IL-17, and IL-12 (p40).

### Determination of Bacterial Loading

Two weeks after the oral *H. pylori* SS1 challenge, stomach tissues were collected to quantify bacterial loading. Lower pathogen loads indicate stronger protection. First, the isolated tissues were washed with cold PBS before transfer to pre-weighed 5-mL tubes containing brain-heart infusion broth (BHI) media. Next, the tubes were re-weighed with tissues to 0.0001-gram accuracy. Tissue fragments were homogenized using a sterile homogenizer and plated serially at dilutions of 1:10, 1:100, and 1:1000 onto Campylobacter Agar Base (Difco) with 10% sheep blood. These were incubated under microaerobic conditions at 37°C for 6–7 days. The colonies were determined to be *Helicobacter* by urease reaction, oxidase reaction, and wet-mount morphological analyses.

### Urease Test

Specimens excised from the ventriculi of each mouse were steeped in 0.5 mL 0.8% NaCl solution and prepared as tissue homogenate, which were used to measure urease activity (Nedrud and Blanchard, 2001). In brief, 3 mL urea broth (1 mg/mL glucose, 1 mg/mL peptone, 2 mg/mL KH_2_PO_4_, 5 mg/mL NaCl, and 1% urea) containing a phenol red indicator was mixed with 100 μL of the tissue homogenate. Tissue homogenate containing PBS only served as a negative control. After incubation at 37°C for 4 h, a UV/vis spectrophotometer was used to determine the urease activity of each gastric tissue (optical density = 550 nm).

### Statistical Analysis

All ELISA experiments were performed in triplicate. To determine significance between the mean values of the experimental and control groups, one-way or two-way analysis of variance (ANOVA) tests were performed and followed by Tukey’s *post hoc* test. All data are expressed as mean ± standard deviation (SD) and analyzed statistically using GraphPad Prism software version five (GraphPad Software Inc., San Diego, California, United States).

## Results

### OMVs Derived From *H. pylori* 7.13 Enhance Both Humoral and Mucosal Immunity

**FIGURE 2:**
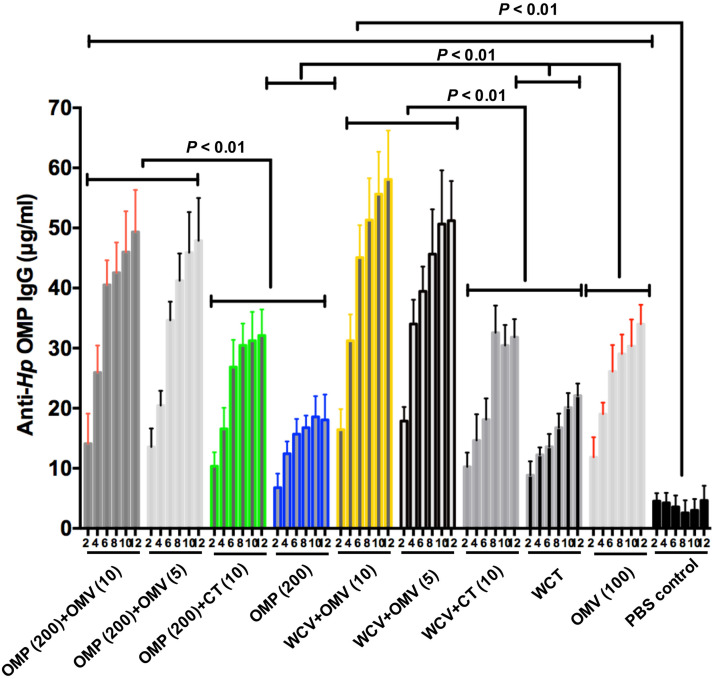
Concentrations of anti-*Hp* OMP IgG within 12 weeks of the first immunization which were quantified by ELISA. Each cohort contained 18 mice. These data reflect IgG levels severally in blood specimen from immunized mice. Group immunized with OMP, WCV, or OMVs derived from *H. pylori* 7.13 served as negative control to discount the interference of vaccine effect. Statistical significance was assessed by two-way ANOVA test. *P* < 0.01 was considered as statistically significant. All the results were expressed as means ± SD per cohort.

To evaluate the effect of OMVs as an adjuvant, two commonly used vaccine candidates, OMPs and WCV, were used. CT was used as the control adjuvant. During immunization, all animals remained in good health and did not exhibit abnormal behavior. The levels of IgG from mice serum were measured by quantitative ELISA. We found that the immune response to 200 μg OMPs was slightly weaker than to WCV, although this difference was not significant (Figure 2). Further, titers of anti-*H. pylori* OMP IgG, which represent the degree of humoral immunity, showed a markedly elevated protective effect with the addition of OMVs to the vaccines (*P* < 0.01) (Figure 2). Moreover, 5 μg OMVs showed greater protective enhancement than 10 μg CT (Figure 2). Furthermore, OMVs as lone antigens induce both humoral and mucosal immune responses more strongly than OMPs and WCV alone (*P* < 0.01) (Figure 2), consistent with previous studies in other pathogens (Vidarsson et al., 2001; Bottero et al., 2016). In addition, the duration of immunity was increased. 12 weeks after the first immunization, all groups supplemented with OMVs as adjuvants sustained a relatively high levels of relevant antibodies, compared to the PBS control group (*P* < 0.01) (Figure 2).

We also determined the levels of secretory IgA (S-IgA) in vaginal secretions, which is the most important indicator of systemic mucosal immunity through vaccination (Kronsteiner et al., 2016; Fan et al., 2018). Analogous phenomenon with the IgG levels could be observed in mucosal immunity, based on anti-*H. pylori* OMP S-IgA (Supplementary Figure 1). Although S-IgA in the vagina may not provide effective protection against *H. pylori* infection in gastric colonization, these results suggested that OMVs as adjuvant could enhance promotion of vaginal mucosal immune responses. This observation expands the scope of applications for OMVs as mucosal adjuvants in vaccine formulations for intestinal or vaginal pathogens.

### Relationship of OMV Components to the Adjuvant Properties

**FIGURE 3:**
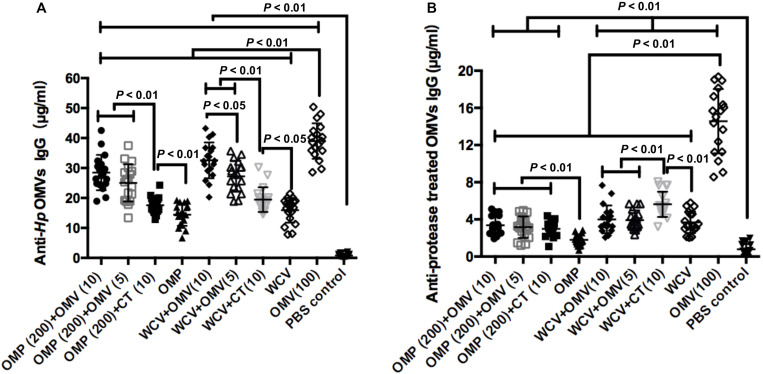
Concentrations of anti-*Hp* OMVs IgG **(A)** and protease treated OMVs IgG **(B)** from mice of all experimental groups were determined quantificationally by ELISA at week 8. The results of IgG analysis are expressed as means ± SD. The means were compared using the least significant difference test. *P* < 0.05 and *P* < 0.01 represents the difference between the related groups.

OMV is composed of OMPs, lipids and lipoproteins. To preliminarily explore which components of OMVs are responsible for the enhanced immune response to the adjuvant, we used the purified OMVs and protease-treated OMVs as the ELISA antigens to measure the IgG levels in the mice sera of all groups at the 8th week. The results showed the vaccines with OMV adjuvant elicit significantly higher anti-*Hp* OMV IgG levels than with CT adjuvant, but lower than OMV immunization (Figure 3A). These data indicated that although OMVs have immunogenicity, they would not interfere with the immune responses to OMP or WCV due to their low dosage. In addition, the data presented in Figure 3B confirms these results. OMVs were cleaved by ultrasound and then incubated with protease, which removes the major protein components while retaining the lipid components, including LPS. The IgG levels of the groups immunized with vaccine plus adjuvant were significantly lower compared to the IgG levels of OMV immunized group (*P* < 0.01). Notably, the IgG levels from WCV plus CT group were significantly higher than the WCV plus OMV group and WCV alone immunized group, and the levels of WCV plus OMV groups were consistent with the WCV alone group (Figure 3B). This suggested that LPS or other lipids did not have an immunogenic role in OMVs as adjuvant. They might play an immunostimulatory role when OMVs are used as an adjuvant to enhance the immunity of antigens, but further experiments are needed to confirm this hypothesis.

### OMVs Derived From *H. pylori* 7.13 Enhance Gastric Mucosal Immunity

**FIGURE 4:**
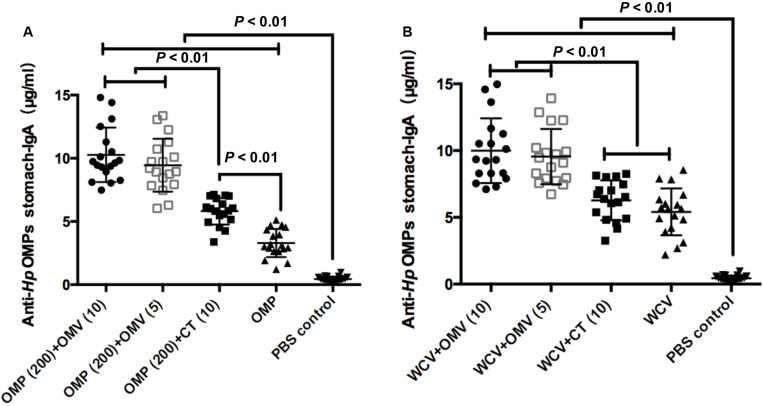
Concentrations of anti-*Hp* OMP stomach-IgA using OMP as vaccine **(A)** or WCV **(B)** from the stomach of mice sacrificed were determined quantificationally by ELISA at week 8. Each group was comprised of 18 mice. These data manifested the strength of mucosal immune response induced by diverse immunogens, which were expressed as means ± SD per group. Least significant difference test was performed to determine whether the distinction between each mean of groups were significant. *P* < 0.01 represents the difference between the related groups.

A previous study reported that the gastric mucosal immune reaction reduces bacterial colonization in the stomach (Nystrom et al., 2006). Therefore, gastric mucosal immunity was analyzed by measuring stomach-IgA in the gastric mucosa isolated from the mice sacrificed at week 8. Results showed that CT could significantly augment the titers of anti-*Hp* OMP stomach-IgA (*P* < 0.01), but no significant increases were found with WCV (*P* > 0.01) (Figures 4A,B). These results are consistent with previous studies that CT is a defective, toxic adjuvant (Wakabayashi et al., 2006; Mattsson et al., 2015). In comparison, OMVs showed better adjuvanticity combined with OMP and WCV as evidenced by the higher titers. The addition of 10 μg or 5 μg OMVs showed significantly higher levels of stomach-IgA than OMP or whole cell antigen (WCT) with 10 μg CT (*P* < 0.01) (Figures 4A,B). This indicated that *H. pylori* 7.13 OMVs might be a better adjuvant than CT in anti-*H. pylori* vaccines.

### *H. pylori* OMVs Strengthen Both Th1 and Th2 Responses

**FIGURE 5:**
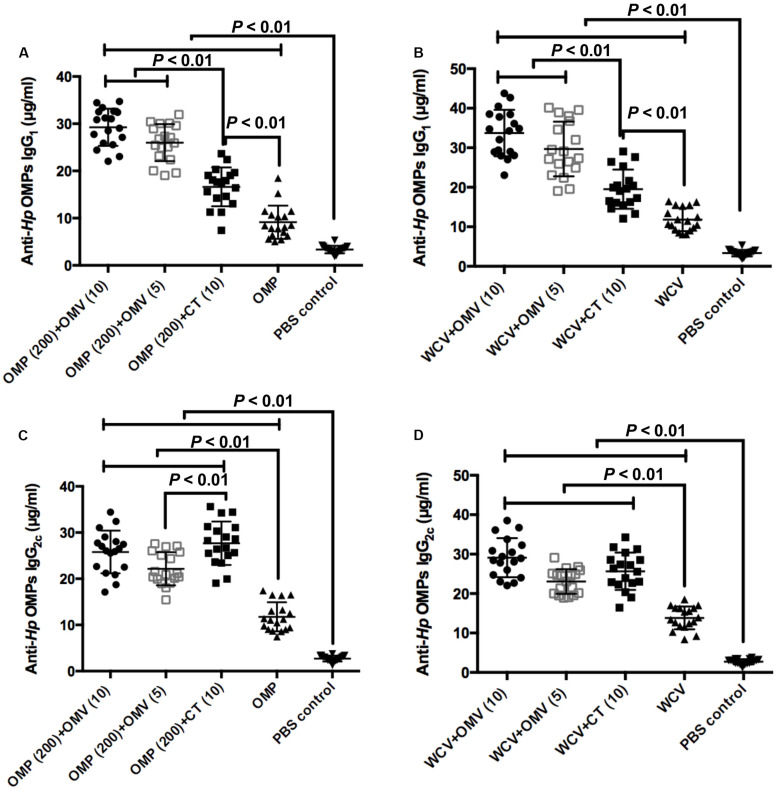
The concentrations of anti-*Hp* IgG_1_ of groups used OMP **(A)** or WCV **(C)** as vaccine, and the levels of anti-*Hp* IgG_2c_ of groups with OMP **(B)** or WCV **(D)** were determined by ELISA against OMPs isolated from *H. pylori*. Each group was comprised of 18 mice. The exact concentration of IgG_1_ and IgG_2c_ subclass antibodies in serum samples of mice at 8 weeks after immunization are shown. The means were compared using the least significant difference test. *P* < 0.01 reflect statistical significance between the related groups.

We measured IgG_1_ and IgG_2__c_ antibody isotypes in blood samples to identify the type of immunity activated by each treatment in mice. T helper type 1 (Th1)-polarized response correlating with IgG_2__c_ in the blood of C57BL/6 mice represents cellular immunity, while the T helper type 2 (Th2) immune response (relating to IgG_1_) represents humoral immunity in mice (Comoy et al., 1997; Martin et al., 1998). To verify the adjuvanticity of OMVs from *H. pylori* 7.13, we compared different vaccine combinations with OMVs, vaccines with CT, and individual vaccines. The levels of anti-*Hp* OMP IgG_1_ in the CT group (10 μg CT + 200 μg OMP or WCV) were significantly higher than the vaccine-only groups (Figures 5A,C) (*P* < 0.01). All groups using OMVs as adjuvants, regardless of amount of OMVs, resulted in superior anti-*Hp* OMP IgG_1_ compared to the CT groups, demonstrating that OMVs augment vaccine-induced humoral immune responses (Figures 5A,B) (*P* < 0.01). In contrast, the concentrations of anti-*Hp* OMP IgG_2__c_ generated by vaccines with OMVs were similar to those generated against CT vaccines, but the concentrations in adjuvant-augmented vaccine groups were still much higher than those in the vaccine-only groups (Figures 5C,D) (*P* < 0.01). There was an apparent enhancement of IgG_2__c_ levels observed in the group immunized with OMP plus CT compared to the group immunized with OMP plus 5 μg OMVs (*P* < 0.01).

**FIGURE 6:**
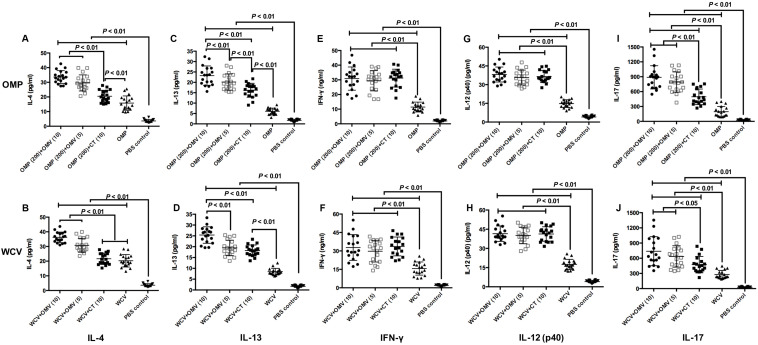
Cytokine productions were detected by excised splenocytes from sacrificed mice at 8 weeks after first immunization. The concentrations of IL-4 **(A,B)**, IL-13 **(C,D)**, IFN-γ **(E,F)**, IL-12 (p40) **(G,H)** and IL-17 **(I,J)** generated by groups used OMP as vaccine **(A,C,E,G,I)** and groups immunized with WCV **(B,D,F,H,J)** were then subjected to cytokine-specific ELISAs. The results of cytokine production analysis are expressed as means ± SD (*n* = 18). Means were compared using the least-significant-difference test. *P* < 0.05 and *P* < 0.01 reflect statistical significance between the related groups.

Additionally, we performed cytokine detection from mice splenocytes to determine the type of immune reaction enhanced by the adjuvant. IFN-γ is secreted by Th1 cells, and IL-12 can facilitate CD4^+^ cells becoming Th1 cells (Comoy et al., 1997). Therefore, these cytokines were regarded as indicators of a Th1-polarized response, whereas IL-4 and IL-13 served as indicators of Th2-polarized response (Prout et al., 2018). In addition, IL-17 mediated Th17 responses play a critical role in vaccine-induced protection against *H. pylori* (Flach et al., 2011). The results showed that OMVs can augment all cytokines, irrespective of the dosage and immunogens, with greater production of IL-4, IL-13 and IL-17 compared to the groups with CT adjuvant (Figure 6) (*P* < 0.01). By contrast, 10 μg CT increases the production of most cytokines compared to the groups immunized with OMP or WCV only (*P* < 0.01), except the level of IL-4 in combination with WCV (Figure 6). All cytokine data demonstrated that OMVs and CT adjuvant had similar activation effect of the Th1 response, but OMVs were more efficient at activating the Th2 and Th17 responses. Doubling the OMVs used significantly increased IL-4 and IL-13 production (Figures 6A–D) (*P* < 0.01), with no corresponding change in IFN-γ, IL-12 and IL-17 (Figures 6E–J) (*P* > 0.01).

Collectively, when *H. pylori* 7.13 OMVs were used as adjuvants, they facilitated both humoral and cellular immunity; however, the response was skewed towards Th2 and Th17 immunity.

### *H. pylori* OMVs Reinforce Vaccine Protection

**FIGURE 7:**
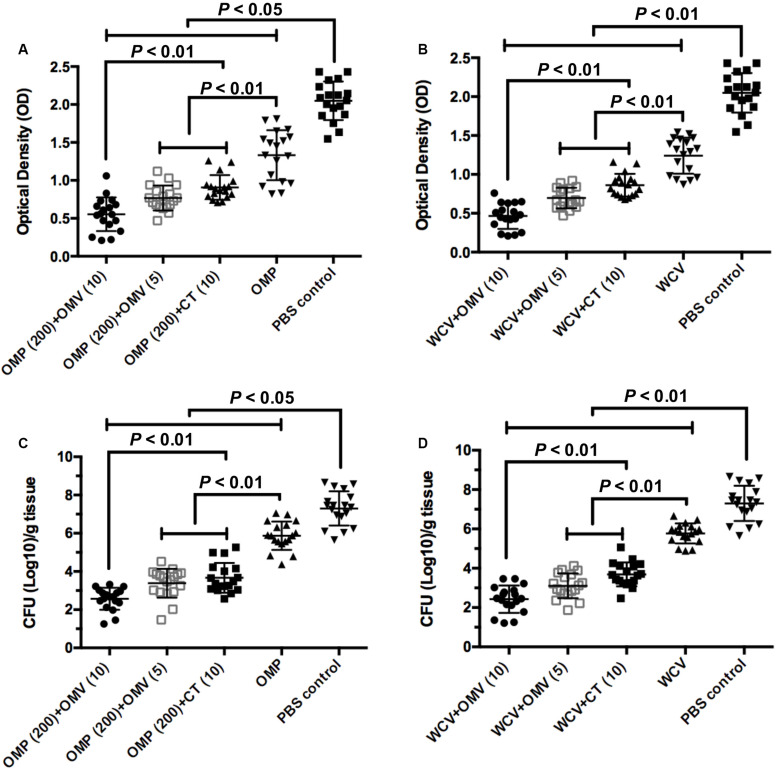
OMVs as adjuvant elicit protection against *H. pylori* SS1 infection. Stomach tissue were collected from immunized mice and washed by PBS buffer to determine the urease activity and bacterial load quantitation. **(A,B)** Urease activity in stomach homogenates from mice immunized OMPs or WCV with OMVs or CT as adjuvant was assessed two weeks after the challenge infection. **(C,D)** *H. pylori* colony counts in stomach homogenates were quantified. The means were compared using the least significant difference test. *P* < 0.05 and *P* < 0.01 reflect statistical significance between the related groups.

UV/vis spectrophotometer was used to measure the density of bacteria in the mouse stomach at the end of rapid urease test. The OD value was positively correlated with urease activity, which can reveal the levels of *H. pylori* colonization in gastric mucosa. OMVs adjuvants with both OMP and WCV decreased the optical density further than the CT versions (Figures 7A,B) (*P* < 0.01). In addition, we cultured *H. pylori* isolated from gastric tissue and counted the CFU to measure the density of the pathogen. The colony counting results were consistent with the OD measurements. The group immunized with 100 μg OMVs had the lowest CFU count of any groups (Figures 7C,D) (*P* < 0.01). In addition, there was no significant difference between 5 μg OMVs and 10 μg CT adjuvants in the same vaccine, showing the effectiveness of OMVs (Figures 7C,D). Both tests verified that OMVs could inhibit the bacteria load in the stomach of C57BL/6 mouse model.

## Discussion

Because some antigens have inadequate immunogenicity and *H. pylori* can evade the host immune response, adjuvants may be useful countermeasures to induce an effective antibody response (Sutton and Chionh, 2013). CT, LT, synthetic oligodeoxynucleotides containing unmethylated CPG motif (CPG-ODN) have been proven efficient as adjuvants. Aluminum hydroxides (Alum) are the most common adjuvants utilized against *H. pylori* in studies, however their enhancement of cell immunity was insufficient (Eisenbarth et al., 2008; Mirzaei et al., 2017). In addition, novel potential adjuvant candidates have been explored, including chitosan and glycoprotein G (gG) from alpha herpes viruses (Gong et al., 2015; Walduck et al., 2015). However, despite these advances, further research is needed to develop suitable adjuvants against *H. pylori* for human use, due to the limitations of current adjuvants.

Our laboratory evaluated for their potential to induce a reliable immune response (Liu et al., 2016, 2019; Tan et al., 2018). OMV-based vaccines against other pathogens such as *N. meningitidis*, *Bordetella pertussis*, and *Acinetobacter baumannii* have been successfully tested in experimental animals (Beernink et al., 2019; Zurita et al., 2019; Pulido et al., 2020). In addition, researchers have demonstrated the potential use of OMVs as adjuvants with the *Neisseria meningitidis* vaccine (Sanders and Feavers, 2011). Recently, we used OMVs derived from *H. pylori* to vaccinate mice and observed favorable results against *H. pylori*, suggesting an OMV-based vaccine as a potential therapeutic treatment against this pathogenic bacterium (Liu et al., 2019). Moreover, due to the lack of a suitable and efficient adjuvant in anti-*H. pylori* vaccines, this study aimed to investigate whether *H. pylori* OMVs could be adjuvants.

When evaluating the effectiveness of a vaccine and adjuvant combination against *H. pylori* infection, the first consideration should be the activation of mucosal immune response, which is closely related to the reduction of gastric bacterial load (Nystrom et al., 2006). The concentration of gastric IgA antibodies against *H. pylori*, representing the degree of gastric mucosal inflammation, was measured by ELISA. The OMV-vaccine combination even elicited a notable enhancement of S-IgA in vaginal secretions, which is further evidence of the outstanding adjuvanticity of OMVs (Supplementary Figure 1). With these results, we hypothesized that OMVs could be used in oral vaccines or against vaginal pathogens.

Additionally, the Th1 and Th2 cytokine profiles of T cell proliferation was assessed (Figures 5, 6). Typically, IgG_1_ and IgG_2__a_ reflect the polarization of the immune response (Comoy et al., 1997). The IgG_2__a_ gene is deleted in C57BL/6 mice, which have an IgG_2__c_ gene instead (Martin et al., 1998). Therefore, the levels of IgG_2__c_ in C57BL/6 mice were used as a measure of Th1 type immune responses. It is widely believed that cellular immunity plays an indispensable role in inhibiting colonization of intracellular pathogens, including *H. pylori*. Cellular immunity is often associated with Th1-, Th17-, or both-specific immunity in mice, although some studies have concluded that Th1 immunity limited clearance from the host (Mohammadi et al., 1996; Taylor et al., 2008; Flach et al., 2011). However, the Th2-polarized immune response (humoral immunity) had a significant role in controlling *H. pylori* infections, which contributed to the generation of *H. pylori*-specific antibodies. Several previous studies demonstrated that Th2 immunity was more important than Th1-cell responses, mainly due to the latter being stimulated in the pathogenic period and causing inflammation (Pan et al., 2018). CT is a standard adjuvant that enhances antigen-specific mucosal and humoral immunity, and promotes a balanced Th1/Th2/Th17 response in vaccine-induced protection (Wakabayashi et al., 2006; Mattsson et al., 2015). In this study, we observed that vaccines with CT indeed triggered a balanced Th1/Th2/Th17-type systematic immune reaction (Figures 5, 6). In contrast, vaccines with OMVs induced a Th2- and Th17-biased immunity against *H. pylori*, which is more efficient in inducing potent systematic and local immune responses (Delyria et al., 2009; Taylor et al., 2008). We also noted that OMVs were more effective in improving the eradication of *H. pylori* infections than CT adjuvant. This indicates that Th2 and Th17 responses play the critical role in protection from *H. pylori.*

Further, the safety of vaccine and adjuvants should be carefully considered. Although VacA has been shown to induce an effective anti-*H. pylori* immune response, its cytotoxicity activity limits its potential development and application to humans (Ki et al., 2009). In our previous study, we tested *H. pylori* OMVs to determine their virulence in mice and concluded they are safe in mice (Liu et al., 2019). This finding showed the potential application of OMVs as a vaccine or adjuvant in humans, with an advantage compared to some toxic adjuvants, such as LPS, chimeric flagellum (CF), and CpG. While use of toxic adjuvants could overcome the low immunogenicity of some vaccines, their apparent or latent risks limit their clinical applications (Luo et al., 2018). In the past decade, several mutant toxins, such as mmCT and double mutant LT, have been developed as both non-toxic and effective mucosal adjuvants for vaccination against *H. pylori* (Sjokvist Ottsjo et al., 2013; Holmgren et al., 2018). However, the production of these mutant toxins require elaborate genetic manipulations, while OMVs were native, non-toxic components which did not need additional detoxification for use.

In addition, availability and storage requirements are important considerations in the design of effective vaccines. While several immunogens are both potent and safe enough for use as a vaccine or adjuvant candidate, complex and costly production limits their clinical use. For example, to produce DNA vaccines, a target DNA fragment must be transferred into a bacterial plasmid, introduced into a bacterial vector via electrotransfection, and then introduced into the bacterial ghost (Chen et al., 2014). Some potential antigen proteins, including OMP and recombinant fusion protein, require plasmid transfection (Voland et al., 2003; Liu et al., 2011). Alpha-galactosylceramide (α-GalCer), an oral adjuvant, was synthesized in a series of reactions (Longet et al., 2019). In order to stimulate OMV production to obtain adequate yields, various stress factors such as temperature, and nutrient depletion or overdosing are controlled and administered during cultivation, which is technically challenging (Klimentova and Stulik, 2015).

OMVs as a specific anti-*H. pylori* adjuvant has two main advantages: safety and efficacy. The results showed that the addition of OMVs could augment both humoral and mucosal immunity, causing a significant attenuation in *H. pylori* colonization. By measuring Th1-, Th2, and Th17-specific cytokines, OMVs of *H. pylori* were shown to induce both responses, favoring Th2 and Th17 immunity. Kuipers et al. reported that OMVs as vaccines displaying heterologous antigens could induce local production of antigen-specific IL-17A, which contributes to the Th17 response (Kuipers et al., 2015, 2017). These findings showed that OMVs strengthen the Th17-biased protection by vaccines through immunostimulatory properties. This induction may be through pathogen-associated molecular patterns (PAMPs) on OMVs that can activate the immune system (Miyaji et al., 2011). In addition, we preliminarily explored the relationship of OMV components to adjuvant properties. The IgG levels of groups immunized with OMPs or WCV plus OMVs against protease-treated OMVs (LPS and other lipids) were significantly lower than the group immunized with OMV alone (Figure 3). Therefore, we could exclude the influence of OMV protein components on the immunity enhancement of OMVs as adjuvants, and hypothesize that OMV adjuvanticity is derived from LPS, which has a substantial role in establishing Th1/Th2 immunity (Taylor et al., 2008). However, this is only a preliminary conclusion and more experiments are needed to clarify which components of OMVs are involved in its adjuvant function.

There were a few limitations in the study. First, isolation and purification of OMVs from *H. pylori* is slightly more rigorous than other immunogens (Klimentova and Stulik, 2015). This may hinder the large-scale use of OMVs. Second, although *H. pylori* LPS is a major pathogenic factor of OMVs, unlike other gram-negative bacteria, the specific structures of *H. pylori* LPS, lipid A and O-polysaccharide antigen can lead to immune escape or autoimmune disease in the host (Moran and Pendergast, 2001; Parker and Keenan, 2012). Therefore, we plan to genetically engineer the structure of *H. pylori* LPS to stimulate the adjuvanticity of OMVs and reduce its toxicity. Finally, we only chose two common vaccines as experimental reagents, hence the compatibility of OMVs as adjuvant with other vaccines requires further study.

## Conclusion

OMVs derived from *H. pylori* as adjuvants enhance Th1/Th2/Th17 immune responses, with a greater effect on Th2 and Th17 immunity, which resulted in better cell-mediated and humoral immunity. Further, gastric colonization of *H. pylori* was attenuated by addition of OMVs, compared to the equivalent CT group, which confirmed that *H. pylori* OMVs could be used as adjuvants in vaccine development.

## Data Availability Statement

The datasets generated for this study are available on request to the corresponding author.

## Ethics Statement

The animal study was reviewed and approved by the Animal Welfare Committee of Nanchang University.

## Author Contributions

QL conceived and designed the experiments. ZS, BL, YZ, RL, HR, and JW performed the experiments. ZS and QL analyzed the data and wrote the manuscript. All authors contributed to the article and approved the submitted version.

## Conflict of Interest

The authors declare that the research was conducted in the absence of any commercial or financial relationships that could be construed as a potential conflict of interest.

## Supplementary Material

FIGURE S1 Concentrations of anti-*Hp* OMP secretory-IgA (S-IgA) within 12 weeks of the first immunization which were quantified by ELISA. Each cohort contained 18 mice. These data reflect S-IgA levels severally in vaginal secretion specimen from immunized mice. Group immunized with OMP, WCV, or OMVs derived from *H. pylori* 7.13 served as negative control to discount the interference of vaccine effect. Statistical significance was assessed by two-way ANOVA test. *P* < 0.01 was considered as statistically significant. All the results were expressed as means ± SD per cohort.
